# Offspring of Mothers With Histories of Chronic and Non-chronic Depression: Symptom Trajectories From Ages 6 to 15

**DOI:** 10.3389/fpsyt.2020.601779

**Published:** 2020-11-19

**Authors:** Jamilah Silver, Thomas M. Olino, Gabrielle A. Carlson, Daniel N. Klein

**Affiliations:** ^1^Department of Psychology, Stony Brook University, Stony Brook, NY, United States; ^2^Department of Psychology, Temple University, Philadelphia, PA, United States; ^3^Department of Psychiatry, Stony Brook University, Stony Brook, NY, United States

**Keywords:** chronic depression, maternal depression, offspring, intergenerational transmission, persistent depression

## Abstract

Several studies have reported that individuals with chronic depression have higher rates of depressive disorders, and particularly chronic depression, in their first-degree relatives, compared to those with non-chronic (episodic) major depression. In addition, a few studies have suggested that offspring of parents with chronic depression have elevated rates of depression and other psychopathology. Most of this work uses the Diagnostic and Statistical Manual of Mental Disorders (DSM), which defines chronicity as persistence for at least 2 years. An alternative is a life-course, approach, which evaluates overall course since first onset. We examined the trajectories of depressive, anxiety, and externalizing symptoms in a community sample of 577 offspring of mothers with histories of chronic depression, non-chronic (or episodic) major depression, and no depression using prospective, multi-informant assessments from age 6 to age 15. Offspring of mothers with a history of depression exhibited higher levels of depression, anxiety, and externalizing symptoms than offspring of mothers who were never depressed. Moreover, the effects of maternal depression on offspring depression, anxiety, and externalizing symptoms were more pronounced for mothers with histories of chronic than non-chronic depression, particularly when the life-course approach to classifying chronicity was used. These data suggest that research that combines chronic and non-chronic depressions includes significant heterogeneity that may hinder understanding of etiology and reduce the likelihood of developing a cumulative and replicable literature. In addition, these findings have significant implications for prevention and treatment.

## Introduction

An extensive body of research has documented the familial aggregation and intergenerational transmission of depressive disorders (1, 2). A smaller group of studies have reported that the relatives of individuals with chronic forms of depression, such as chronic major depression or dysthymic disorder, referred to as “persistent depressive disorder” in the Diagnostic and Statistical Manual of Mental Disorders, 5th edition [DSM-5, (3)] experience even higher rates of depressive disorders than relatives of persons with non-chronic, or episodic, major depression (4–7). In addition to being at greater risk for depression in general, relatives of people with chronic depression have higher rates of chronic depression than relatives of individuals with non-chronic major depression, suggesting some degree of specificity of familial aggregation (5, 6, 8).

There has been particular interest in examining the development of depression in the offspring of depressed parents, especially depressed mothers, as this is a high risk group that offers critical opportunities for prevention and early intervention, as well as for examining risk factors and mechanisms prior to the onset of the disorder (2). Studies of clinical and community samples indicate that the rate of depressive disorders in offspring of depressed parents is 2–3 times greater than in the offspring of non-depressed parents (9–12). Moreover, this increase in risk is particularly pronounced among offspring of depressed mothers (13). Children of mothers with depressive disorders are also at increased risk for anxiety, behavior, and substance disorders (11, 14, 15).

Paralleling the literature on the familial aggregation of chronic depression, a handful of studies have reported that the children of chronically depressed parents are at even higher risk than offspring of non-chronically depressed parents. Compared to offspring of non-chronically depressed mothers, offspring of chronically depressed mothers have higher rates of depression, recurrent major depressive episodes, and chronic depression (4, 10). Moreover, offspring of chronically depressed mothers also exhibit higher rates of behavioral problems and substance use disorders (4, 16, 17).

However, a problem plaguing the literature on chronic depression is the wide variation in how chronicity is defined (18). While many studies classify chronic depression using the DSM, which itself has changed across editions, a number define chronicity on the basis of mean scores on repeated assessments of self-reported (19) or interviewer-rated (17) symptoms.

The most widely-used approach in the literature on chronic depression utilizes DSM-III, DSM-III-R, and DSM-IV categories and episode and course specifiers (20–22), which were combined under the rubric of Persistent Depressive Disorder in DSM-5 (3). The DSMs define chronicity as a duration of at least 2 years, which, while reasonable, is arbitrary and is not based on evidence (23). This approach may be most useful for clinical samples, when patients present in a current episode. However, in non-clinical samples, it is possible for individuals to have past episodes that meet DSM criteria for chronic depression while still having a life course that is relatively depression-free (e.g., an individual who experienced a 2-year period of dysthymia at age 30, followed by full recovery and no recurrences over the next 20 years) (23). Hence, Mondimore et al. (24) suggested an alternative approach to defining chronicity using a life course perspective, where chronicity is evaluated on the basis of the individual's course since their first onset of depression. Mondimore et al. (24) reported that this approach exhibited good interrater reliability (Kappa = 0.76). Applying Robins and Guze (25) classic framework for validating psychiatric diagnoses, they compared the familial aggregation of chronic depression using the lifetime and DSM approaches to defining chronic depression. Mondimore et al. (24) found that their approach yielded higher levels of familial aggregation, as indexed by the odds of a relative exhibiting chronic depression, than the DSM approach.

In the present study, we examine the trajectories of depressive, anxiety, and externalizing symptoms in a community sample of offspring of mothers with histories of chronic depression, non-chronic (or episodic) major depression, and no depression using prospective, multi-informant assessments conducted every 3 years from age 6 to age 15. In addition to defining chronic and non-chronic depression using DSM-IV, we conducted parallel analyses using a life-course approach to classifying chronic and non-chronic depression (24). Based on previous literature, we expected that offspring of depressed mothers would exhibit higher levels of depressive symptoms over the course of childhood into adolescence than offspring of never-depressed mothers, and that these effects would be most pronounced among offspring of mothers with a history of chronic depression. We also expected that offspring of depressed mothers would exhibit higher levels of anxiety and externalizing symptoms, and that these effects would be particularly prominent among the offspring of mothers with chronic depression. Finally, given preliminary evidence that a life-course approach to defining chronicity may enhance the differences in familial aggregation between chronic and non-chronic depression compared to DSM classification (24), we conjectured that the hypothesized effects would be more pronounced when chronic and non-chronic depression are classified using a life-course perspective.

## Methods

### Participants

Families with a 3-year-old child living within 20 miles of Stony Brook, NY were recruited using commercial mailing lists for a larger study of risk for emotional disorders; children with significant medical or developmental disorders were excluded, and the child had to live with at least one biological parent [*N* = 559; (26)]. An additional 50 families were added in the second wave of assessments when children were 6 years old in order to increase the diversity of the sample. Only one child per family was included in the study. The sample was re-assessed when children were ~9, 12, and 15 years old. Retention rates in waves 2–5 were 84.4, 81.3, 80.0, and 76.7%, respectively.

The current analysis sample included 577 children and mothers. Participants were included if the child's mother completed a diagnostic interview about her own history of psychopathology as part of her initial assessment. The mean ages of the children in the analysis sample at each wave were 3.56 years (SD = 0.26; range: 2.92–4.17), 6.08 years (SD = 0.41; range: 4.83–7.57), 9.18 years (SD = 0.39; range: 8.33–10.92), 12.66 years (SD = 0.46; range: 11.50–14.17), and 15.25 years (SD = 0.40; range: 14.43–17.64), respectively. Of the children in the analytic sample, 265 (45.8%) were female, 522 (90.5%) were white, 37 (6.4%) were Black, 14 (2.4%) were Asian, 1 (0.2%) were Native American, and 3 (0.5%) were other. Sixty seven (11.6%) offspring were Hispanic. Due to the small Ns, we coded race/ethnicity as white and non-Hispanic (*N* = 469; 81.3%) or as non-white and/or Hispanic (*N* = 108; 18.7%). More than half of children (379 [69.7%]) had at least one parent who had graduated from college at the initial assessment. The demographic characteristics of the sample were representative of the surrounding county (27).

### Measures

#### Maternal Depression

The Structured Clinical Interview for DSM–IV non-patient version (SCID) was used to assess maternal history of depression (28) at the age 3 (or age 6 for the 50 additional families) wave. As part of the interview, we obtained a detailed follow-back timeline of the course of depression from the DSM-IV Mood Disorders Field Trials (29, 30). The SCID was administered by telephone to 577 mothers by a highly experienced masters-level clinician. Based on audiotapes of 30 randomly selected interviews, interrater reliability (kappa) for lifetime depressive disorders was 0.93. In previous studies, our interviewer demonstrated high interrater reliability for distinguishing DSM-IV chronic and non-chronic depression and rating clinical course using the timeline (31, 32).

For the present study, we defined chronic and non-chronic (or episodic) depression in mothers in two different ways: using the DSM-IV (22) and applying a life course perspective (24). Using DSM-IV, 386 (66.9%) mothers were never depressed, 112 (19.4%) mothers had lifetime non-chronic (or episodic) major depressive epiosde, and 79 (13.7%) mothers had chronic depression (chronic major depressive episode and/or dysthymic disorder).

Using the DSM approach, 48 (60.8%) mothers with chronic depression had co-morbid anxiety and 64 (57.1%) mothers with non-chronic depression had co-morbid anxiety. Twenty six (32.9%) mothers with chronic depression had co-morbid substance abuse and 37 (33.0%) mothers with non-chronic depression had co-morbid substance abuse. Finally, 1 (1.3%) mothers with chronic depression and 1 (0.9%) mother with non-chronic depression had co-morbid bipolar disorder. The two groups did not significantly differ on co-morbid anxiety (*X*^2^ = 0.250, *p* = 0.617), substance abuse (*X*^2^ = 0.000, *p* = 0.986), or bipolar disorder (*X*^2^ = 0.062, *p* = 0.803).

Using a life course perspective, mothers' depression was categorized based on course from the initial episode of depression to the time of the SCID assessment. Non-chronic depression was defined as: (1) a single episode of depression lasting up to 2 years (*N* = 68; 35.6%), (2) recurrent episodes of depression lasting up to 2 years with significant (>6 mos) periods of interepisode recovery (*N* = 26, 13.6%), (3) recurrent episodes of depression lasting up to 2 years with brief periods ( ≤ 6 mos) of interepisode recovery (*N* = 1; 0.05%), or (4) one or more episodes of chronic (>2 years) depression but with total time in remission longer than total time depressed since onset (*N* = 44; 23.0%). Chronic depression was defined as: (1) chronic (>2 years) depression with total time in remission shorter than total time depressed since onset (*N* = 27; 14.1%) or (2) mostly or virtually always depressed and never well for >2 consecutive months (*N* = 25; 13.1%). In sum, our life course criteria classified 386 (66.9%) mothers as never depressed, 139 (24.1%) mothers as having a history of non-chronic depression, and 52 (9.0%) mothers as having a history of chronic depression.

With regard to the life course approach, 29 (55.8%) mothers with chronic depression had co-morbid anxiety and 83 (59.7%) mothers with non-chronic depression had co-morbid anxiety. Nineteen (36.5%) mothers with chronic depression had co-morbid substance abuse and 44 (31.7%) mothers with non-chronic depression had co-morbid substance abuse. Finally, no mothers with chronic depression and 2 (1.4%) mothers with non-chronic depression had co-morbid bipolar disorder. The two groups did not significantly differ on co-morbid anxiety (*X*^2^ = 0.243, *p* = 0.622), substance abuse (*X*^2^ = 0.408, *p* = 0.523), or bipolar disorder (*X*^2^ = 0.756, *p* = 0.385).

#### Early Childhood, Middle Childhood and Adolescent Symptoms

At the age 6, 9, 12, and 15 waves, children, and at the age 9, 12, and 15 waves, mothers and fathers, completed the child- and parent-report versions of the Children's Depression Inventory [CDI; (33)], a measure of depressive symptoms during the past 2 weeks that is designed for youth aged 7–17. At age 6, items were read aloud to the children. Cronbach's alpha across the waves ranged from 0.74–0.82 for child reports, 0.78–0.80 for mother reports, and 0.76–0.79 for father reports.

At the age 9, 12, and 15 waves, children, mothers, and fathers also completed the 41-item child- and parent-report versions, respectively, of the Screen for Childhood Anxiety Related Disorders [SCARED; (34)], a measure of anxiety symptoms over the past 3 months designed for youth aged 9–18. The SCARED is made up of five factor-analytically derived subscales: panic/somatic, generalized anxiety, separation anxiety, social phobia, and school phobia. In the current sample, Cronbach's alpha across the waves ranged from 0.89 to 0.93 for child reports, 0.90 to 0.91 for mother reports, and 0.88 to 0.89 for father reports.

Finally, at the age 6, 9, 12, and 15 waves, mothers and fathers completed the CBCL 6–18 (35). In the present paper, we examine the broadband internalizing (32 items) and externalizing (35 items) scales. For the internalizing scale, Cronbach's alpha across the waves ranged from 0.86 to 0.87 for mothers and 0.78 to 0.90 for fathers. Alphas for the externalizing scale ranged from 0.87 to 0.88 for mothers and 0.87 to 0.91 for fathers.

#### Data Analyses

Multilevel models were used to test the associations between maternal depression and CDI depression, SCARED anxiety, and CBCL internalizing and externalizing symptoms across assessment waves. In these models, time was centered at the final assessment, so the intercept reflects the level of the dependent variable at age 15. The models included both random intercept and random slope components. Time was coded as wave number, and missing data were estimated using Maximum Likliehood Estimation (ML). Multilevel models were conducted using Mplus (36); all other statistical analyses were performed using SPSS 25 (37).

## Results

Using DSM-IV, offspring of chronically, non-chronically, and never depressed mothers did not differ on race/ethnicity, *X*^2^(2, *N* = 577) = 2.86, *p* = 0.24, sex, *X*^2^ (2, *N* = 577) = 2.69, *p* = 0.26, age at baseline, *F*_(2,538)_ = 0.66, *p* = 0.52, or having at least one parent who had graduated from college, *X*^2^ (2, *N* = 577) = 1.59, *p* = 0.45. Similarly, using the life-course approach, children of chronically, non-chronically, and never depressed mothers did not differ on race/ethnicity, *X*^2^ (2, *N* = 577) = 4.02, *p* = 0.13, sex, *X*^2^ (2, *N* = 577) = 1.41, *p* = 0.49, age at baseline, *F*_(2,538)_ = 0.42, *p* = 0.65, or having at least one parent who had graduated from college, *X*^2^ (2, *N* = 577) = 3.14, *p* = 0.20.

The life-course approach to defining chronicity was narrower than the approach in DSM-IV. Of the 112 mothers with non-chronic major depression using DSM-IV, 107 (95.5%) had non-chronic depression and 5 (4.5%) had chronic depression using the life-course approach. Of the 79 mothers with chronic depression using DSM-IV, 48 (60.8%) had chronic depression and 31 (39.2%) had non-chronic depression using the life-course perspective.

### Associations of DSM-IV Maternal Chronic Depression With Child Symptoms

Means of each symptom measure at each wave can be seen in Table 1. Correlations between measures are presented in Supplementary Table 1; in line with the literature, these correlations are generally moderate in magnitude.

**Table 1:** N, Means, and SDs of offspring symptom variables across assessment waves.

	**Age 6**		**Age 9**		**Age 12**		**Age 15**	
**Measure**	***N***	***M* (SD)**	***N***	***M* (SD)**	***N***	***M* (SD)**	***N***	***M* (SD)**
Child CDI	485	7.42 (5.27)	467	4.80 (4.16)	456	4.82 (5.33)	442	5.66 (5.30)
Mother CDI	-	-	471	7.20 (4.83)	461	7.09 (5.00)	447	8.02 (5.46)
Father CDI	-	-	414	7.35 (4.42)	374	7.50 (5.02)	370	8.03 (5.05)
Child SCARED	-	-	466	19.46 (11.02)	458	16.66 (10.57)	440	17.10 (12.08)
Mother SCARED	-	-	470	7.92 (8.03)	455	7.91 (7.90)	447	6.65 (7.60)
Father SCARED	-	-	412	6.72 (6.56)	372	7.02 (6.85)	369	6.04 (6.66)
Mother CBCL internalizing	455	3.54 (4.58)	471	4.06 (4.87)	460	3.63 (4.87)	447	3.78 (5.13)
Father CBCL internalizing	363	3.69 (3.81)	413	3.74 (4.96)	374	4.03 (5.09)	370	3.91 (5.80)
Mother CBCL externalizing	455	5.14 (5.69)	471	4.56 (5.27)	460	3.51 (4.75)	447	3.16 (4.50)
Father CBCL externalizing	363	5.44 (5.66)	413	4.45 (5.55)	374	4.40 (5.14)	447	3.16 (4.50)

*CDI, Children's Depression Inventory; SCARED, Screen for Childhood Anxiety Related Disorders; CBCL, Children's Behavior Checklist*.

The first set of analyses used DSM-IV to define chronic and non-chronic depression. First, multilevel models were run to estimate the associations of maternal depression with the linear effect of time (i.e., assessment wave) on child, mother, and father-reports of child depressive symptoms on the CDI (see Table 2). Compared to mothers who had never been depressed, maternal non-chronic major depression predicted the intercept of mother-reported depressive symptoms in offspring. Offspring of non-chronically depressed mothers exhibited significantly higher estimated levels of mother-reported depressive symptoms at the final wave than offspring of never-depressed mothers. Similarly, offspring of chronically depressed mothers exhibited significantly higher estimated levels of both mother- and child-reported depressive symptoms at the age 15 wave than offspring of never-depressed mothers. Finally, compared to mothers with non-chronic depression, offspring of chronically depressed mothers exhibited significantly higher estimated levels of mother-reported depressive symptoms at the final wave.

**Table 2:** Multi-level models using maternal depression at age 3 to predict symptom outcomes across subsequent waves using the DSM approach.

	**Non-chronic vs. Never depressed**		**Chronic vs. Never depressed**		**Chronic vs. Non-chronic**	
	**Intercept**	**Slope**	**Intercept**	**Slope**	**Intercept**	**Slope**
**Measure**	***B* (SE)**	***B* (SE)**	***B* (SE)**	***B* (SE)**	***B* (SE)**	***B* (SE)**
Child CDI	0.03 (0.62)	−0.02 (0.28)	2.45 (0.69)^**^	0.45 (0.32)	2.41 (0.84)^**^	0.48 (0.38)
Mother CDI	1.62 (0.62)^**^	0.18 (0.30)	3.19 (0.69)^**^	0.15 (0.34)	1.56 (0.83)	−0.03 (0.40)
Father CDI	0.63 (0.65)	0.01 (0.33)	1.09 (0.75)	−0.05 (0.38)	0.45 (0.89)	−0.07 (0.46)
Child SCARED	1.65 (1.46)	0.79 (0.88)	3.40 (1.61)^*^	0.71 (0.98)	1.74 (1.95)	−0.07 (1.18)
Mother SCARED	2.46 (0.91)^**^	−0.43 (0.44)	3.87 (1.01)^**^	0.33 (0.49)	1.40 (1.25)	0.76 (0.59)
Father SCARED	−0.50 (1.15)	−1.05 (0.42)^*^	−0.18 (1.31)	−0.35 (0.48)	−0.38 (1.18)	0.70 (0.59)
Mother CBCL internalizing	1.78 (0.60)^**^	0.01 (0.22)	2.98 (0.67)^**^	−0.05 (0.22)	1.23 (0.81)	−0.06 (0.30)
Father CBCL internalizing	2.04 (0.72)^**^	0.26 (0.25)	−0.85 (0.99)	−0.46 (0.34)	1.19 (0.82)	−0.20 (0.28)
Mother CBCL externalizing	0.57 (0.52)	−0.02 (0.21)	1.53 (0.58)^**^	−0.57 (0.23)^*^	0.95 (0.70)	−0.54 (0.28)
Father CBCL externalizing	1.34 (0.70)	0.28 (0.27)	0.89 (0.79)	−0.38 (0.30)	−0.45 (0.95)	−0.66 (0.37)

** *p < 0.01*,

* *p < 0.05*.

*CDI, Children's Depression Inventory; SCARED, Screen for Childhood Anxiety Related Disorders; CBCL, Children's Behavior Checklist*.

**Figure 1:**
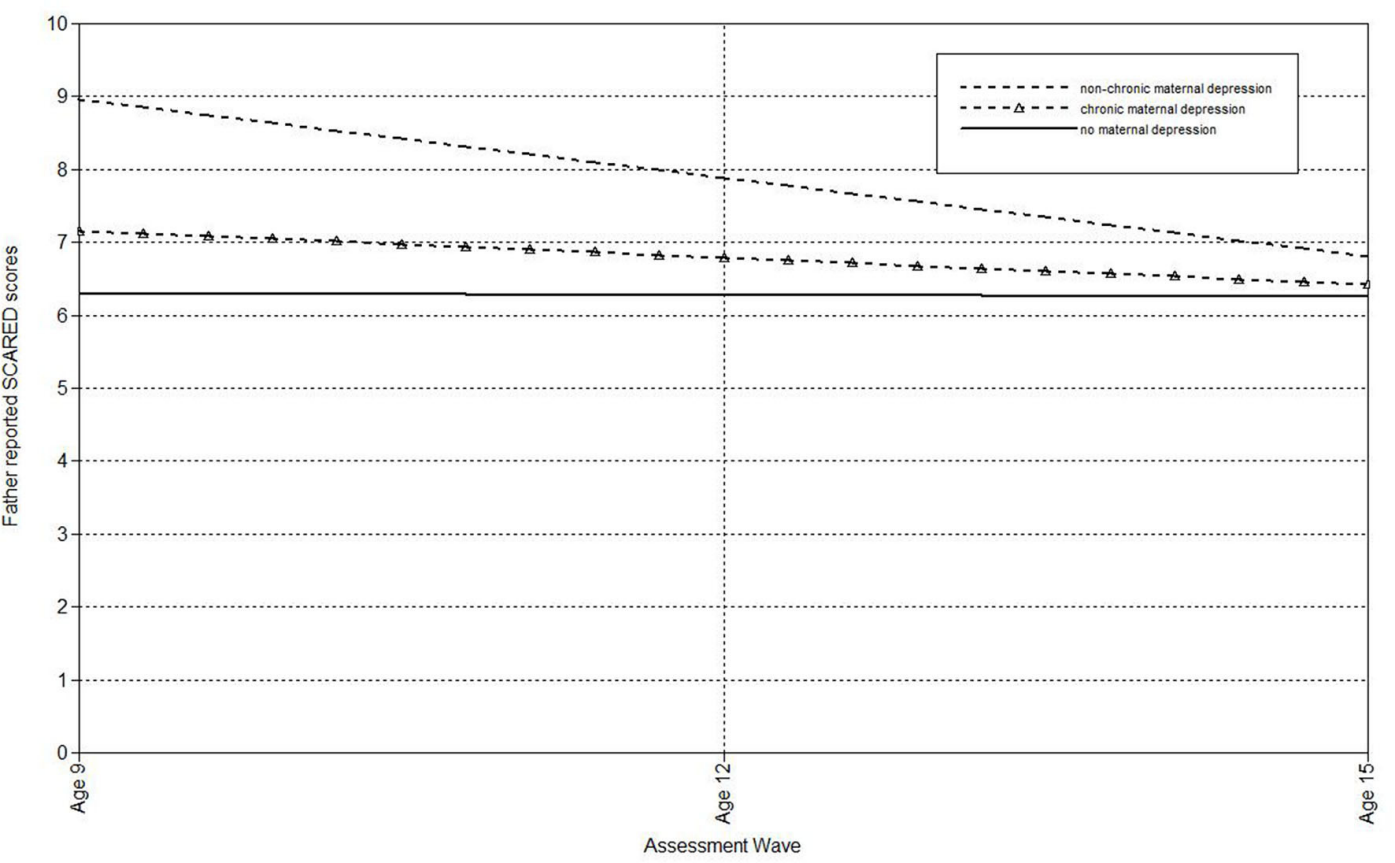
Effect of non-chronic maternal depression (DSM approach) on the slope of father-reported SCARED scores in offspring.

Next, multilevel models were run to estimate the associations of maternal depression with the linear effect of time on child, mother, and father-reports on the SCARED (see Table 2). Compared to mothers who had never been depressed, offspring of non-chronically depressed mothers exhibited higher estimated levels of mother-reported anxiety symptoms at the final wave. Additionally, maternal non-chronic depression predicted the slope of father-reported anxiety symptoms in offspring. Figure 1 shows the trajectory of change in father-reported SCARED scores in offspring of mothers with no depression, non-chronic major depression, and chronic depression. In this figure, maternal non-chronic depression was associated with more rapid declines in father-reported anxiety symptoms over time. Compared to mothers who had never been depressed, offspring of mothers with chronic depression exhibited higher estimated levels of both child- and mother-reported anxiety symptoms at the age 15 assessment. Offspring of mothers with chronic and non-chronic depression did not differ on the intercepts or slopes of SCARED scores regardless of informant.

Multilevel models were also run to estimate the associations of maternal depression with the linear effect of time on mother- and father-reported CBCL internalizing scores in offspring (Table 2). Offspring of non-chronically depressed mothers exhibited significantly higher estimated levels of both mother- and father-reported internalizing symptoms at the final wave than offspring of never-depressed mothers. Similarly, offspring of mothers with chronic depression exhibited higher estimated levels of mother-reported internalizing symptoms at the age 15 assessment than offspring of never-depressed mothers. Offspring of chronically and non-chronically depressed mothers did not differ on the intercept or slope of mother- or father-reported CBCL internalizing scores.

**Figure 2:**
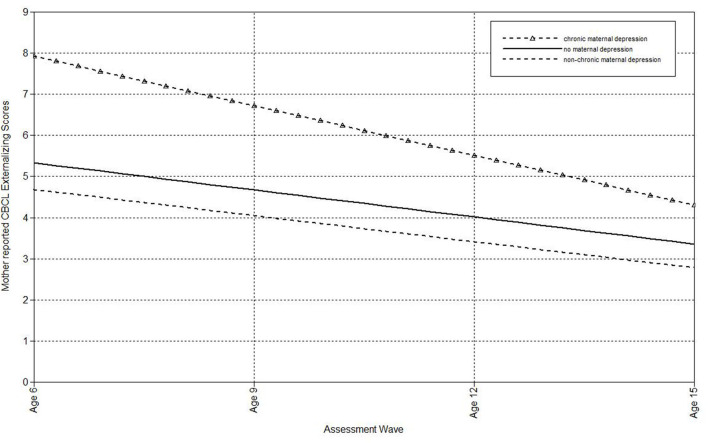
Effect of chronic maternal depression (DSM approach) on the slope of mother-reported CBCL Externalizing scores in offspring.

Finally, multilevel models were run to estimate the associations of maternal depression with the linear effect of time on mother- and father-reported CBCL externalizing scores in offspring (Table 2). Offspring of non-chronically and never depressed mothers did not differ on the intercepts or slopes of mother- or father-reported externalizing symptoms. Compared to mothers who had never been depressed, maternal chronic depression predicted both the intercept and slope of mother-reported CBCL externalizing scores. As shown in Figure 2, children of mothers with chronic depression exhibited more rapid declines in externalizing symptoms over time, but still continued to have higher estimated externalizing scores at the final wave than offspring of never-depressed mothers. Offspring of chronically and non-chronically depressed mothers did not differ on intercepts or slopes of mother- or father-reported CBCL externalizing scores.

### Associations of Life-Course Maternal Chronic Depression With Child Symptoms

**Figure 3:**
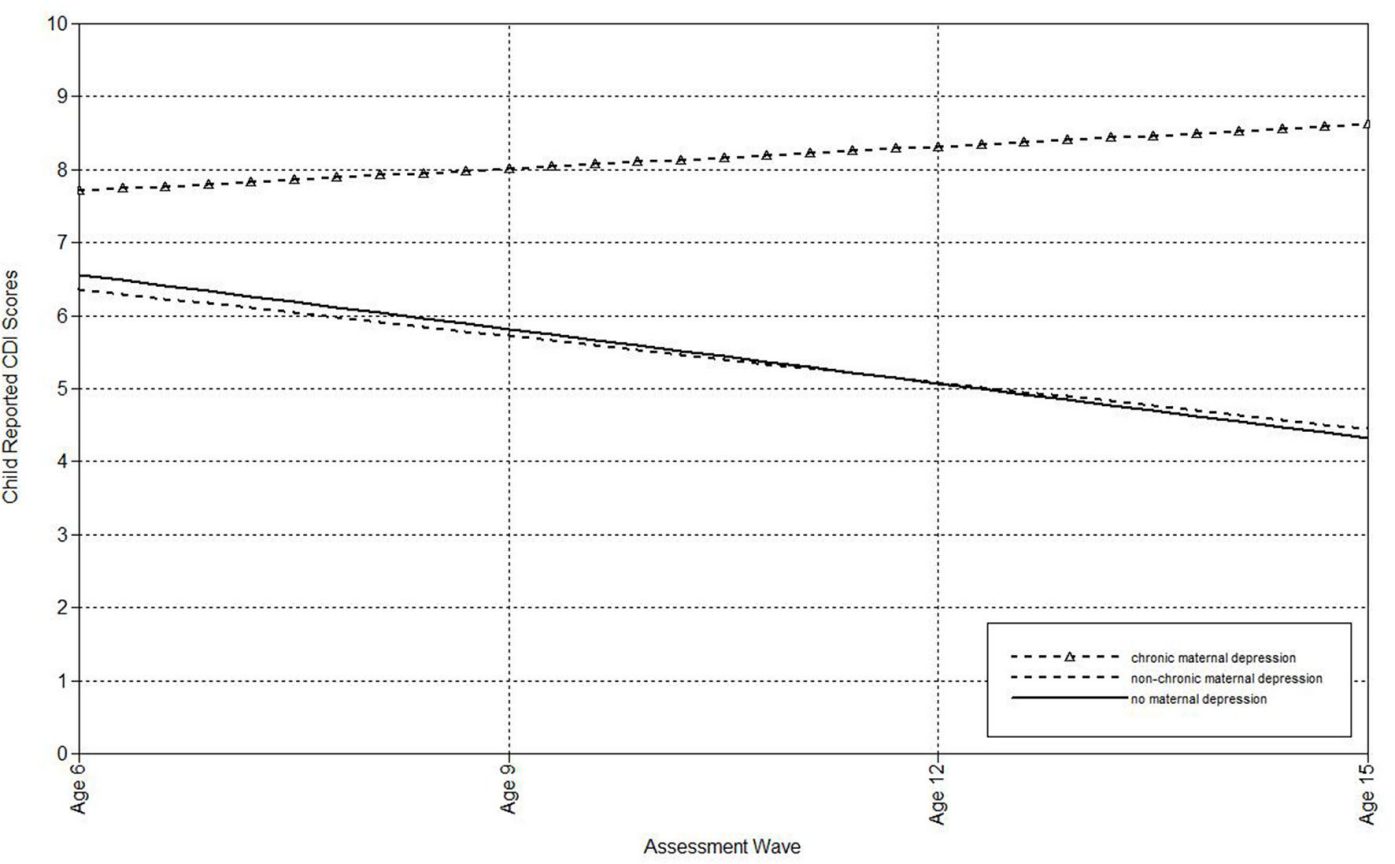
Effect of chronic maternal depression (life-course perspective) on the slope of child-reported CDI scores in offspring.

**Figure 4:**
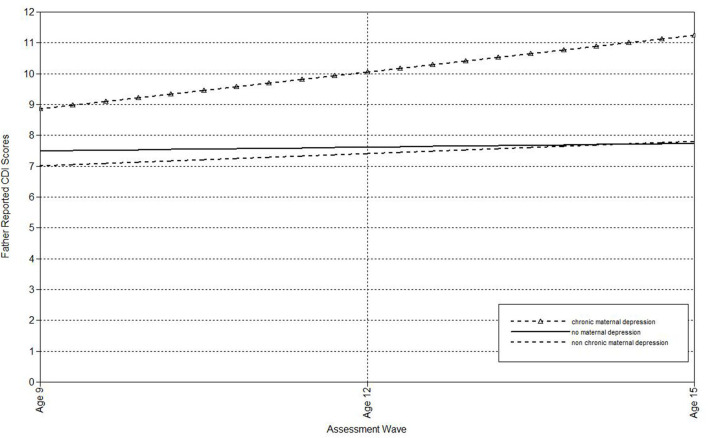
Effect of chronic maternal depression (life-course perspective) on the slope of father-reported CDI scores in offspring.

The second set of analyses used the life-course approach to classifying chronic and non-chronic depression. First, multilevel models were run to estimate the associations of maternal depression with the linear effect of time (i.e., assessment wave) on child-, mother-, and father-reports on the CDI (see Table 3). Offspring of non-chronically depressed mothers exhibited significantly higher estimated levels of mother-reported depressive symptoms at the final wave than offspring of never-depressed mothers. Compared both to mothers who had never been depressed and to mothers with non-chronic depression, maternal chronic depression predicted the intercepts of child-, mother-, and father-reported depressive symptoms in offspring, as well as the slopes of child-reported CDI scores. In addition, compared to mothers with non-chronic depression, maternal chronic depression predicted the slopes of father-reported depressive symptoms (Table 3). In each of these comparisons, the intercept effects indicated that offspring of mothers with chronic depression exhibited significantly higher estimated levels of depressive symptoms at the final assessment. In addition, offspring of mothers with chronic depression exhibited a more rapid increase in child-reported depression symptoms over time compared to offspring of both mothers with non-chronic depression and those with no history of depression (Figure 3), as well as a more rapid increase in father-reported CDI scores than offspring of mothers with non-chronic depression (Figure 4).

**Table 3:** Multi-level models using maternal depression at age 3 to predict symptom outcomes across subsequent waves using the life course approach.

	**Non–chronic vs. Never depressed**		**Chronic vs. Never depressed**		**Chronic vs. Non-chronic**	
	**Intercept**	**Slope**	**Intercept**	**Slope**	**Intercept**	**Slope**
**Measure**	***B* (SE)**	***B* (SE)**	***B* (SE)**	***B* (SE)**	***B* (SE)**	***B* (SE)**
Child CDI	0.12 (0.56)	0.10 (0.26)	4.17 (0.82)^**^	0.94 (0.38)^*^	4.29 (0.91)^**^	1.04 (0.42)*
Mother CDI	−1.22 (0.55)^*^	−0.01 (0.27)	5.08 (0.82)^**^	0.56 (0.41)	3.85 (0.90)^**^	0.54 (0.45)
Father CDI	0.07 (0.58)	0.27 (0.30)	3.3 (0.91)^**^	0.79 (0.48)	3.50 (1.00)^**^	0.10 (0.52)^*^
Child SCARED	−0.40 (1.32)	0.03 (0.80)	7.51 (1.92)^**^	2.81 (1.20)^*^	7.10 (2.12)^**^	2.84 (1.31)^*^
Mother SCARED	−1.70 (0.82)^*^	0.43 (0.40)	6.65 (1.21)^**^	0.74 (0.60)	4.95 (1.33)^**^	1.17 (0.65)
Father SCARED	0.32 (0.78)	1.02 (0.38)^**^	2.45 (1.21)^*^	0.03 (0.61)	2.78 (1.32)^*^	1.05 (0.66)
Mother CBCL internalizing	−1.36 (0.54)^*^	0.05 (0.20)	4.63 (0.80)^**^	0.08 (0.30)	3.26 (0.88)^**^	0.14 (0.34)
Father CBCL internalizing	−0.93 (0.66)	0.02 (0.23)	3.76 (1.01)^**^	0.30 (0.36)	2.82 (1.11)^*^	0.33 (0.40)
Mother CBCL externalizing	−0.24 (0.45)	0.27 (0.19)	2.83 (0.69)^**^	−0.20 (0.28)	2.58 (0.77)^**^	0.07 (0.31)
Father CBCL externalizing	−0.56 (0.63)	−0.05 (0.24)	2.74 (0.98)^**^	−0.17 (0.39)	2.18 (1.08)^*^	−0.22 (0.42)

** *p < 0.01*,

* *p < 0.05*.

*CDI, Children's Depression Inventory; SCARED, Screen for Childhood Anxiety Related Disorders; CBCL, Children's Behavior Checklist*.

**Figure 5:**
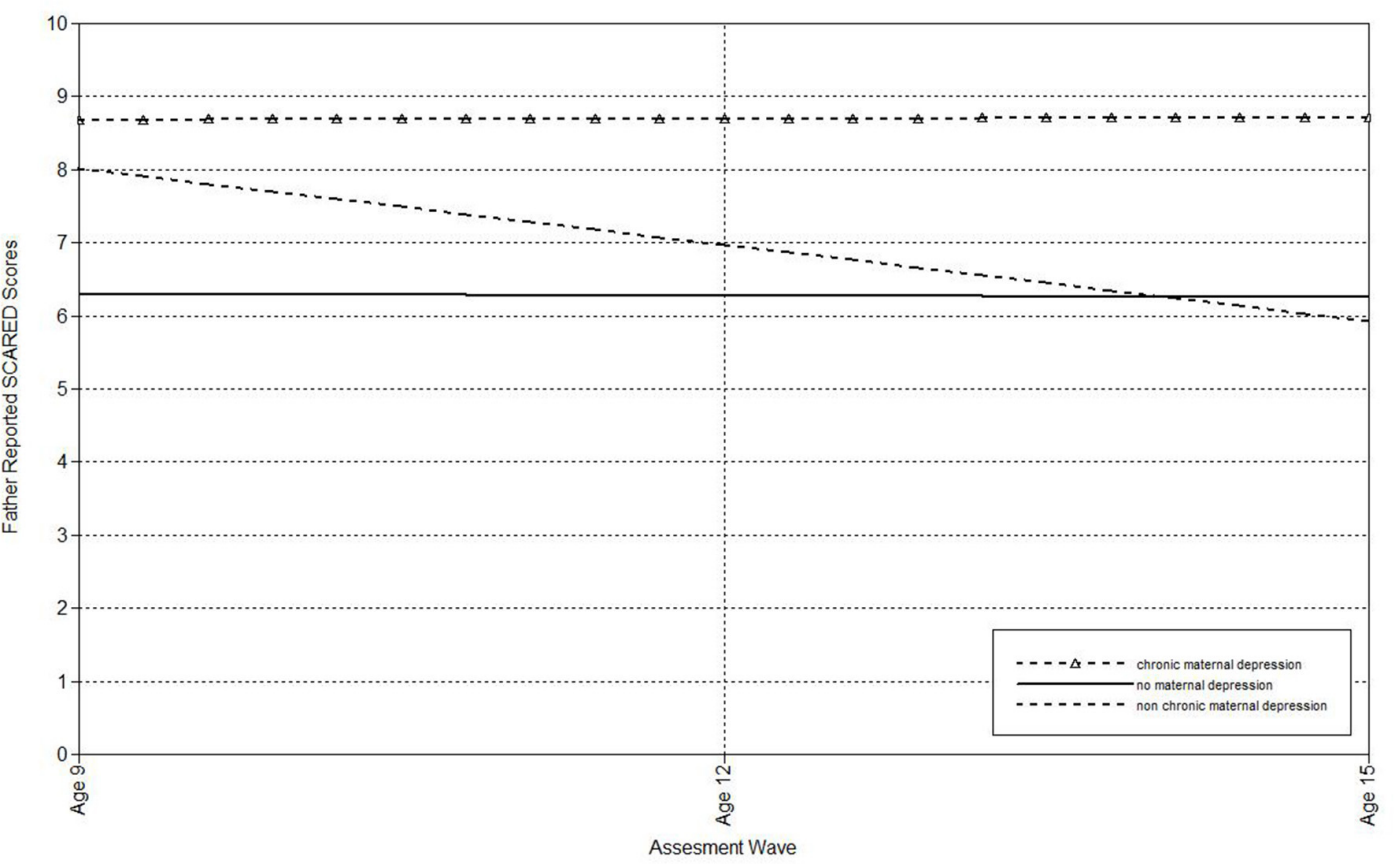
Effect of non-chronic maternal depression (life-course perspective) on the slope of father-reported SCARED scores in offspring.

**Figure 6:**
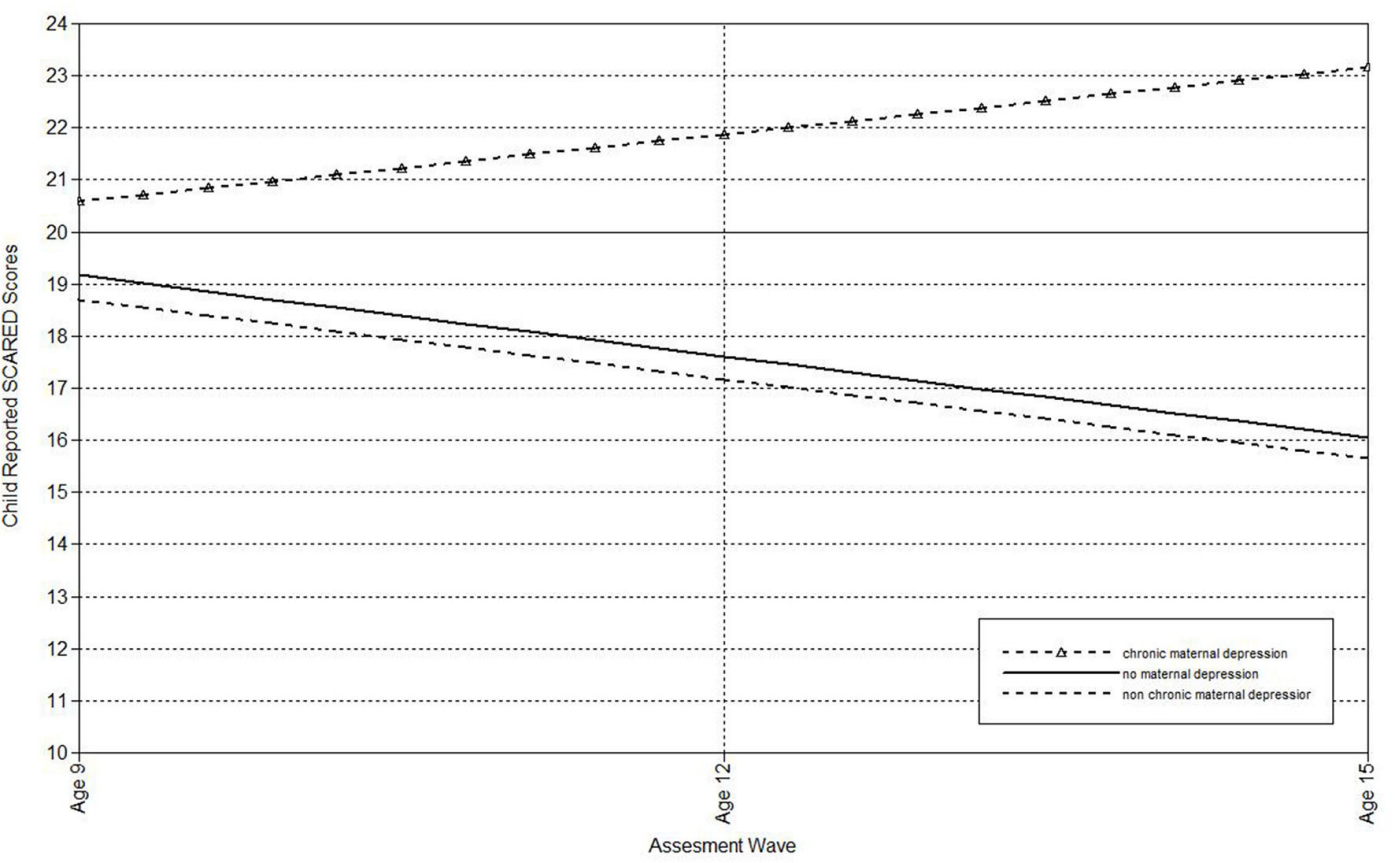
Effect of chronic maternal depression (life-course perspective) on the slope of child-reported SCARED scores in offspring.

Next, multilevel models were run to estimate the associations of maternal depression with the linear effect of time on child-, mother-, and father-reports on the SCARED (Table 3). Offspring of non-chronically depressed mothers exhibited significantly higher estimated levels of mother reported anxiety symptoms at the last wave than offspring of never-depressed mothers. Non-chronic depression in mothers also predicted the slope of father-reported SCARED scores. Offspring of mothers with non-chronic depression exhibited more rapid declines in father-reported anxiety symptoms over time (Figure 5). Compared both to mothers who had never been depressed and mothers with a history of non-chronic depression, maternal chronic depression predicted higher estimated levels of child-, mother-, and father-reported SCARED scores in offspring at the age 15 wave (Table 3). Moreover, maternal chronic depression also predicted the slope of child-reported SCARED scores (Figure 6). Offspring of mothers with chronic depression exhibited more rapid increases in child-reported anxiety symptoms over time than offspring of both mothers with non-chronic depression and mothers with no history of depression.

Multilevel models were also run to estimate the associations of maternal depression with the linear effect of time on mother- and father-reported CBCL internalizing scores (Table 3). Offspring of non-chronically depressed mothers exhibited significantly higher estimated levels of mother-reported internalizing symptoms at the final assessment than offspring of never-depressed mothers. In addition, offspring of mothers with chronic depression exhibited higher estimated levels of internalizing symptoms, as reported by both parents, at the final assessment than children of both never-depressed and non-chronically depressed mothers.

Finally, multilevel models were run to estimate the associations of maternal depression with the linear effect of time on mother- and father-reported CBCL externalizing symptoms (Table 3). Children of mothers with chronic depression exhibited significantly higher estimated levels of externalizing problems at the final assessment, according to reports from both parents, than offspring of both non-depressed and non-chronically depressed mothers.

## Discussion

The present study examined the trajectories of depressive, anxiety, and externalizing symptoms in a community sample of offspring of mothers with histories of chronic depression, non-chronic (or episodic) major depression, and no depression using prospective, multi-informant assessments conducted every 3 years from age 6 to age 15. In addition to defining chronic and non-chronic depression using DSM-IV, we conducted parallel analyses using a life-course approach to classify chronic and non-chronic depression.

As expected, offspring of mothers with a history of depression generally exhibited higher estimated levels of depression, anxiety, and externalizing symptoms at the final assessment, a period when the increase in depression begins a rapid ascent (38). These findings echo the large literature documenting the familial aggregation (1) and intergenerational transmission of depressive disorders (9–12), as well as the many previous studies reporting that children of mothers with depressive disorders are also at increased risk for anxiety and behavioral disorders (2, 11, 14, 15).

However, a more nuanced picture emerged when the distinction between chronic and non-chronic depression in mothers was considered, and particularly when the DSM-IV and life-course approaches to defining chronicity were examined. Our findings generally supported the hypothesis that the effects of maternal depression on offspring are more pronounced for mothers with histories of chronic than non-chronic depression, although these effects were considerably stronger using the life-course approach to classifying chronicity.

Using the DSM-IV approach to classifying chronicity, comparisons of offspring of chronically depressed and never depressed mothers revealed a somewhat greater number of significant intercept effects (on 6 of 10 measures) than comparisons of offspring of non-chronically depressed and never depressed mothers (on 4 of 10 measures). In all cases, offspring of depressed mothers had significantly higher estimated levels of symptoms in the final assessment. In the only significant slope effects, offspring of non-chronically depressed mothers exhibited significantly faster decreases in father-reported anxiety symptoms and offspring of chronically depressed mothers exhibited significantly faster decreases in mother-reported externalizing symptoms than offspring of never-depressed mothers, suggesting that the effects of maternal depression on anxiety and externalizing symptoms faded over time. In direct comparisons of offspring of mothers with chronic vs. non-chronic depression, the children of mothers with chronic depression reported significantly higher estimated levels of depressive symptoms at the final assessment but did not differ on other 9 intercept and 10 slope comparisons.

However, when chronicity was classified using a life-course approach, offspring of mothers with chronic depression differed from offspring of never-depressed mothers on the intercepts for each of the ten symptom measures examined (reflecting higher estimated levels of depression, anxiety, and externalizing symptoms, as reported by offspring, mothers, and fathers, at the final assessment). In addition, there were two significant slope effects, with offspring of chronically depressed mothers reporting significantly greater increases in depression and anxiety symptoms over time. In contrast, when offspring of non-chronically depressed and never depressed mothers were compared using the life-course approach there were only two significant intercept effects, and one significant slope effect reflecting a faster decrease in father-reported anxiety. Most telling were the direct comparisons between the offspring of mothers with histories of chronic and non-chronic depression. In contrast to the single significant intercept effect observed with DSM-IV-defined groups, when the life-course approach was used, the offspring of chronically depressed mothers exhibited significantly higher intercepts for all ten symptom measures (across three domains of symptoms and three informants) examined, as well as three significant slope effects. The slope effects revealed that offspring of chronically, compared to non-chronically, depressed mothers exhibited significantly greater increases in child- and father-reported depression and child-reported anxiety symptoms over time. Notably, these were the only instances in which a group of offspring showed progressively increasing levels of symptoms over the course of the four follow-up waves and suggest that these youth are already exhibiting signs of chronicity.

Taken together, these findings support the limited prior literature suggesting that the offspring of chronically depressed mothers are at even greater risk for depression and other forms of psychopathology than offspring of non-chronically depressed mothers (4, 10, 17), as well as evidence of the specificity of familial aggregation of chronic depression (4–6, 8). These data are also consistent with previous suggestions that a life-course approach to defining chronic depression may have greater validity than the approach currently adopted in the DSM (24).

In our community sample, the life-course approach to defining chronic depression was considerably narrower than the DSM-IV approach. Almost all participants (96%) who met DSM-IV criteria for non-chronic major depression were also classified as having non-chronic depression with the life-course approach. The few exceptions were cases that exhibited persistent depressive symptoms for more than half the time since the onset of depression, but did not quite meet full DSM-IV criteria for chronic major depressive episode or dysthymia (e.g., occasional periods of remission of >2 months that precluded the latter diagnosis). However, only 61% of those classified as having chronic depression by DSM-IV were also classified as having chronic depression using the life-course approach. The discrepant cases almost always involved lengthy (>2 years) but time-limited depressive episodes in the context of a largely depression-free course since onset. Thus, from a life-course perspective, a significant number of cases of DSM-IV chronic depression are actually episodic conditions, albeit with prolonged episodes. As the DSM-5 category of persistent depression places the DSM-IV chronic depressive conditions under a single rubric, the results using DSM-5 would probably be quite similar. However, we suspect that the DSM and life-course approaches to defining chronic depression would show much greater concordance in clinical samples, as most cases will be presenting with chronic depression and their future course is unknown.

The present study did not address which factors are responsible for the greater psychopathology in the offspring of mothers with chronic, compared to non-chronic, depression. A number of factors have been implicated in the intergenerational transmission of depression (2), some or all of which may account for the greater risk to offspring of mothers with chronic depression, including greater or different genetic liability, a higher rate of parental personality disorder, more problematic parenting, and higher levels of familial and peer stress (39).

The findings from the present study have a number of important implications. First, consistent with prior work [e.g., (18, 32)], these data suggest that the predominant approach to research on depressive disorders, which ignores the course of depression and combines chronic and episodic cases, includes significant heterogeneity that may hinder understanding of etiology and pathophysiology and reduce the likelihood of developing a cumulative and replicable literature. In addition, these findings have significant implications for nosology and assessment. The DSM has given increasingly greater recognition to the importance of longitudinal course in classifying depressive disorders in the last two editions (18, 29), but the present findings suggest that further efforts are needed. They also highlight the challenge faced by alternative nosological systems, such as the Hierarchical Taxonomy of Psychopathology (40), which has the virtue of being empirically-derived, but as of yet has not been able to incorporate a longitudinal perspective into its cross-sectional taxonomy (18). One approach, proposed by Klein (23), is to classify depression using two orthogonal axes representing symptom severity and longitudinal course. This approach has the advantage of capturing the primary depression diagnoses, but incorporating them within a dimensional framework.

The present findings also underscore the need for greater attention to longitudinal course in designing structured and semi-structured diagnostic interviews and rating scales (18, 30). Furthermore, our findings have implications for prevention and early intervention, as they suggest that it may be more efficient to target chronically depressed parents and their offspring, rather than depressed parents more generally. Finally, these results highlight the potential value of developing treatments specifically designed to target chronic, as opposed to all, forms of depression [e.g., (41)].

### Strengths and Limitations

This study had a number of strengths. The sample was relatively large; we compared two different approaches to defining chronicity of maternal depression; offspring's trajectories were assessed on 4 occasions at 3-year intervals from age 6 to age 15; and we collected data on offspring's symptoms from multiple informants. We chose to use multiple informants due to the known limitations of single informants in general, and of self-reports and parent reports, specifically (42). Use of multiple informants who vary in their access to different types of symptoms and the specific contexts in which they observe behavior provides a more comprehensive perspective and reduces the effects of rater biases. Notably, in the present study few results differed by informant, providing greater confidence in the robustness of the effects.

However, a number of limitations must be acknowledged. First, the study relied on mothers' retrospective reports of their histories of depression, and the reliability of such reports may be modest. Second, we used DSM-IV, rather than DSM-5 criteria for mothers' diagnoses. However, the criteria for non-chronic major depression were not altered in DSM-5, and, as noted above, the major change in classifying chronic depression involved grouping the several forms of DSM-IV chronic depression under the rubric of persistent depressive disorder (18). Third, interrater reliability of lifetime chronicity in our study is not available, although Mondimore et al. (24) reported good reliability.

Fourth, we focused on offspring's symptoms, rather than diagnoses, as by age 15 offspring were just entering the period when rates of depressive disorders begin to increase, and there were not yet a large enough number of diagnosable cases to allow for robust analyses. While dimensional approaches are often preferable to categorical diagnoses because of their greater reliability and statistical power (23, 40), our results are likely capturing the early development of depression, and analyses of diagnosable cases must await the next wave of follow-ups. In addition, future research that includes functional outcomes would be useful.

Fifth, it is also important to acknowledge that many of the same youth who experienced elevated levels of depression symptoms also had elevated levels of anxiety and externalizing symptoms. Hence, our findings on outcomes are not independent of one another.

Sixth, we examined mothers' histories of depression only prior to the baseline assessment. Thus, children's outcomes may reflect exposure to their mothers' continued depression, rather than simply the effects of maternal depression prior to the initial assessment. More specifically, we cannot determine whether the persisting or increasing symptoms in offspring of chronically depressed mothers (particularly when defined according to the life-course approach) are associated with the continued persistence of their mothers' depression, or whether offspring's symptoms persist even when mothers recover from chronic depression. In future studies it will be important to examine the association between the course of maternal depression and the trajectories of symptoms in their offspring.

Seventh, the sample was largely Caucasian and middle class, and as such, results should be replicated in more diverse populations. Finally, we focused exclusively on depressed mothers given evidence that maternal depression has stronger effects on offspring than paternal depression (13). However, it would be worthwhile for future studies to extend this work by including depressed fathers.

## Conclusions

Overall, this study provides support for the effects of maternal depression, and more specifically, maternal chronic depression, on offspring's risk for depression, anxiety, and externalizing symptoms. Given the potential long-term effects of maternal chronic depression on offspring, early identification, appropriate treatment, and follow-up of depressed women and their children should be a key priority. Finally, the fact that effects were more pronounced when depression was classified using a life-course perspective has critical implications for the larger literature on depression and underscores an important source of heterogeneity that may be better captured from a life-course, rather than the traditional DSM, approach (23, 24).

## Data Availability Statement

The raw data supporting the conclusions of this article will be made available by the authors, without undue reservation.

## Ethics Statement

The studies involving human participants were reviewed and approved by Stony Brook University Committee on Research Involving Human Subjects. Written informed consent to participate in this study was provided by the participants' legal guardian/next of kin.

## Author Contributions

JS helped conceptualize the study, conducted the analyses, and wrote the initial drafts of the manuscript. TO helped design and interpret the analyses and contributed to revising the manuscript. GC helped interpret the findings and contributed to revising the manuscript. DK helped conceptualize the study and design the analyses, designed and obtained finding for the larger project that the data are derived from, and contributed to revising the manuscript. All authors contributed to the article and approved the submitted version.

## Conflict of Interest

The authors declare that the research was conducted in the absence of any commercial or financial relationships that could be construed as a potential conflict of interest. The handling editor declared a past co-authorship with one of the authors DK.
