# Supplementary material for: Offspring of Mothers With Histories of Chronic and Non-chronic Depression: Symptom Trajectories From Ages 6 to 15
Source: Front Psychiatry. 2020 Nov 19;11:601779. doi: 10.3389/fpsyt.2020.601779 (PMC7710605; doi:10.3389/fpsyt.2020.601779)
Supplement: Supplementary file 1 [file Table_1.docx]

**Supplementary Table 1: Within-informant and within-wave correlations between measures of offspring psychopathology**

| **Mothers** | **Age 6** | **Age 9** | **Age 12** | **Age 15** |
| --- | --- | --- | --- | --- |
| CDI with SCARED | - | .42 | .44 | .47 |
| CDI with CBCL-INT | - | .58 | .59 | .60 |
| CDI with CBCL-EXT | - | .59 | .60 | .62 |
| SCARED with CBCL-INT | - | .75 | .78 | .79 |
| SCARED with CBCL-EXT | - | .38 | .39 | .35 |
| CBCL-INT with CBCL-EXT | .58 | .54 | .55 | .52 |
| **Fathers** |  |  |  |  |
| CDI with SCARED | - | .47 | .45 | .41 |
| CDI with CBCL-INT | - | .51 | .61 | .50 |
| CDI with CBCL-EXT | - | .53 | .62 | .51 |
| SCARED with CBCL-INT | - | .62 | .75 | .61 |
| SCARED with CBCL-EXT | - | .37 | .36 | .25 |
| CBCL-INT with CBCL-EXT | .58 | .71 | .59 | .71 |
| **Children** |  |  |  |  |
| CDI with SCARED | - | .43 | .59 | .62 |

CDI = Children’s Depression Inventory; SCARED = Screen for Childhood Anxiety Related Disorders; CBCL = Child Behavior Checklist; INT = Internalizing problems; EXT = Externalizing problems.
